# Anti-spike S1 IgA, anti-spike trimeric IgG, and anti-spike RBD IgG response after BNT162b2 COVID-19 mRNA vaccination in healthcare workers

**DOI:** 10.5937/jomb0-32373

**Published:** 2021-09-03

**Authors:** Gian Luca Salvagno, Brandon M. Henry, Giovanni di Piazza, Laura Pighi, Simone de Nitto, Damiano Bragantini, Gian Luca Gianfilippi, Giuseppe Lippi

**Affiliations:** 1 University of Verona, Section of Clinical Biochemistry, Verona, Italy; 2 Pederzoli Hospital, Service of Laboratory Medicine, Peschiera del Garda, Italy; 3 Cincinnati Children's Hospital Medical Center, The Heart Institute, Cincinnati, Ohio, United States of America; 4 Pederzoli Hospital, Medical Direction, Peschiera del Garda, Italy; 5 Pederzoli Hospital, Infectious Diseases Unit, Peschiera del Garda, Italy

**Keywords:** COVID-19, vaccination, immune response, antibodies, IgA, COVID-19, vakcinacija, imuni odgovor, antitela, IgA

## Abstract

**Background:**

Most studies on immune response after coronavirus disease 2019 (COVID-19) vaccination focused on serum IgG antibodies and cell-mediated immunity, discounting the role of anti-SARS-CoV-2 neutralizing IgA antibodies in preventing viral infection. This study was aimed to quantify serum IgG and IgA neutralizing antibodies after mRNA COVID-19 vaccination in baseline SARS-CoV-2 seronegative healthcare workers.

**Methods:**

The study population consisted of 181 SARSCoV-2 seronegative healthcare workers (median age 42 years, 59.7% women), receiving two doses of Pfizer COVID-19 vaccine BNT162b2 (Comirnaty). Serum samples were collected before receiving the first vaccine dose, 21 days (before the second vaccine dose) and 50 days afterwards. We then measured anti-spike trimeric IgG (Liaison XL, DiaSorin), anti-spike receptor binding domain (RBD) IgG (Access 2, Beckman Coulter) and anti-spike S1 subunit IgA (ELISA, Euroimmun). Results were presented as median and interquartile range (IQR).

**Results:**

Vaccine administration elicited all anti-SARS-CoV2 antibodies measured. Thirty days after the second vaccine dose, 100% positivization occurred for anti-spike trimeric IgG and anti-spike RBD IgG, whilst 1.7% subjects remained anti-spike S1 IgA negative. The overall increase of antibodies level ratio over baseline after the second vaccine dose was 576.1 (IQR, 360.7-867.8) for anti-spike trimeric IgG, 1426.0 (IQR, 742.0-2698.6) for anti-spike RBD IgG, and 20.2 (IQR, 12.5-32.1) for anti-spike S1 IgA. Significant inverse association was found between age and overall increase of anti-spike trimeric IgG (r=-0.24; p=0.001) and anti-spike S1 IgA (r=-0.16; p=0.028), but not with anti-spike RBD IgG (r=-0.05; p=0.497).

**Conclusions:**

mRNA COVID-19 vaccination elicits sustained serum levels of anti-spike trimeric IgG and anti-spike RBD IgG, while also modestly but significantly increasing those of anti-spike S1 IgA.

## Abstract (sr)

**Uvod:**

Većina studija imunog odgovora nakon oboljenja korona virusom (COVID-19) i vakcinacije zasniva se na serumskim IgG antitelima i ćelijski posredovanom imunitetu, ne vodeći računa o anti-SARS-CoV-2 neutralizujućim IgA antitelima i sprečavanju virusne infekcije. Ovo izučavanje ima za cilj da kvantifikuje serumski IgG i IgA neutralizujućih antitela nakon mRNA COVID-19 vakcinacije kod SARSCOV-2 seronegativnih zdravstvenih radnika.

**Metode:**

Populaciju za izučavanje činilo je 181 SARS-COV-2 seronegativna zdravstvena radnika (oko 42 godine starosti, 59,76% su bile `ene) koji su primili dve doze Pfizer COVID10 vakcinu BNT162b2 (Comirnaty). Uzorci seruma su uzeti pre prijema prve vakcine, 21 dan pre druge doze vakcine i 50 dana nakon druge doze vakcine. Mereni su anti-spike trimerik IgG (Liaison XL, DiaSorin), anti-spike receptor vezujući domen (RBD), IgG (Access 2, Beckman Cooulter) i antispike S1 subjedinica IgA (ELISA, Euroimmun). Rezultati su prikazani kako medijana i interkvartilna oblast (IQR).

**Rezultati:**

Vakcina je proizvela sva anti-SARS-CoV-2 antitela koja su merena. Trideset dana nakon davanja doze vakcine javila se 100% pozitivnost na anti-spike trimerni IGG i antispike RBS IgG, dok je 1,7% osoba bilo negativno na antispike S1 IgA. Ukupno povećanje nivoa antitela iznad bazične granice nakon druge vakcine bila je 576,1 (IQR, 360,7-867,8) za anti-spike trimerni IgG, 1426,0 (IQR, 742,0-2698,6) za anti-spike RBS IgG i 290,2 (IQR, 12,5-32,1) za anti-spike S1 IgA. Značajno obrnuta povezanost je nađena između godina i i ukupnog povećanja anti-spike trimernog IgG (r = -0,24; p = 0,001) i anti-spike S1 IgA (r = 0,16; p = -0,028) ali ne i sa anti-spike RBD IgG (r= 0,05; p = 0,497).

**Zaključak:**

mRNA COVID-19 vakcinacija dovodi do povećanja nivoa u serumu anti-spike trimernog IgA i RBD IgG, kao i do značajnog povećanja u serumu anti-spike S1 IgA.

## Introduction

After the first case of severe acute respiratory syndrome coronavirus disease 2019 (SARS-CoV-2) infection has been described in the Chinese city of Wuhan, at the end of the 2019 [1], the severe and multifaceted infectious disease caused by this novel coronavirus spread across the world, achieving pandemic dimensions. SARS-CoV-2 mainly spreads with two different mechanisms, encompassing airborne transmission through inhalation of infected droplets or aerosols, as well as by direct contact with infected objects or surfaces followed by transportation of viable viral particles to eyes, nose or mouth, where they penetrate the host cells through biding to its natural receptor, the angiotensin converting enzyme 2 (ACE-2) [2]. According to these paradigmatic modes of transmission, many mitigation measures have been endorsed to prevent or limit the risk of SARS-CoV-2 contagion, such as face covering, hand hygiene, social distancing, contact tracing and quarantine [3]. Though these measures, both alone and in combination, were proven effective to mitigate the community spread of SARS-CoV-2, the burden of patients with coronavirus disease 2019 (COVID-19) remains dramatically high worldwide [4], such that alternative and even more radical strategies should be envisaged.

According to the World Health Organization (WHO), vaccination is considered a simple, secure and effective means for safeguarding the population against the risk of contracting infectious diseases or developing severe forms of infection-related illness [5]. In practice, vaccine administration uses the host natural defenses (i.e., the immune system) for developing resistance against a specific microorganism though development of three principal mechanisms, i.e., production of specific antibodies (humoral immunity), training of immune cells to produce immune mediators, to kill the pathogen and/or the infected host cells (cellular immunity), as well as forming the so-called memory cells, which could be effective to contrast recurrent or repeated infections caused by the same pathogen.

The current generation of COVID-19 vaccines encompass four major formulations, including lipid-based mRNA-lipid nanoparticles vaccines (mRNA-LNPs), vector (mostly adenovirus-based) DNA vaccines, attenuated or inactivated viruses, along with protein-based subunit vaccines [6]. Compared to other types of vaccines currently approved for use in humans, mRNA-LNPs vaccines have several advantages such as their natural ability to reproduce the infection without deliverance of viable viral particles (i.e., the mRNA is delivered into the host cells, where is translated into antigen viral proteins), the potentially lower immunogenicity and cytotoxicity, the possibility to contain multimeric antigens (so enabling rapid reengineering), the lower need for adjuvants for boosting the immune response, along with the greater potential to be manufactured quickly, at higher volumes and lower costs [7]. These favorable characteristics have paved the way to rapid and massive commercialization of mRNA-LNPs, which are now available worldwide for human use in two brands, marketed by Pfizer (COVID-19 mRNA vaccine BNT162b2; Comirnaty) and Moderna (COVID-19 mRNA vaccine mRNA-1273; Moderna COVID 19 Vaccine).

Several lines of evidence now concur to attest that the ongoing vaccination campaign is associated with high protection against development of severe COVID-19 illness (i.e., between 90-100% reduction for the two mRNA-LNPs), and moreover, may also be effective at decreasing viral circulation, by reducing the risk of SARS-CoV-2 infection in vaccinated individuals (i.e., 94-95% lower for the two mRNA-LNPs) [8]. The substantial reduction in the risk of developing severe COVID-19 illness after vaccination seems mostly attributable to development of circulating neutralizing antibodies, mostly of the IgG class, which specifically target the spike protein of SARS-CoV-2, its S1 subunit or its receptor binding domain (RBD), and thereby limit or completely abolish the binding with host natural viral receptors (i.e., mostly ACE2) [9]. Unlike this well-established mechanism, prevention of SARS-CoV-2 infection seems instead attributable to mucosal immunity, which is essentially based on the presence of primed immune cells and secretory antibodies of the IgA and IgG class, which would be effective to rapidly neutralize the virus at the mucosal surface, thus impeding or completely abolishing host penetration and spread to target organs [10]. With the exception of a few preliminary case series and reports, characterized by relatively limited sample sizes [11] [12] [13], most studies that have explored, deciphered and monitored the immune response developed after COVID-19 vaccination have almost solely focused on assaying serum IgG antibodies and systemic cell-mediated immunity [14], thus discounting the potential role that neutralizing IgA antibodies would play in limiting viral spread in the community and thereby facilitating the achievement of herd immunity.

This study was hence aimed at assessing the humoral immune response developing after mRNA COVID-19 vaccination in a sample of baseline SARS-CoV-2 seronegative healthcare workers, with assessment of antibodies of both IgG and IgA classes, targeting the entire SARS-CoV-2 Spike protein trimer (anti-spike trimeric IgG), the RBD (anti-spike RBD IgG) or the S1 subunit (anti-spike S1 IgA), and thus reliably reflecting the neutralizing antibodies potential developing after vaccination.

## Materials and Methods

### Study population

Our study population consisted of a cohort of SARS-CoV-2 seronegative healthcare workers of the Pederzoli Hospital of Peschiera del Garda (Verona, Italy), who voluntary accepted to be vaccinated with the mRNA-LNPs COVID-19 vaccine BNT162b2 (Comirnaty; Pfizer-BioNTech, NY, USA). Baseline seronegativity was confirmed the day before vaccination via routine monitoring with a Roche Elecsys Anti-SARS-CoV-2 S immunoassay, performed on a Roche Cobas 6000 (Roche Diagnostics, Basel, Switzerland; seronegativity defined as having anti-SARS-CoV-2 S total antibodies levels <0.8 U/L). A first dose of 30 μg of vaccine was administered between January 4 and 7, 2021, which was then followed by the second dose of 30 μg vaccine administered exactly 3 weeks after the first. All vaccines were prepared following manufacturer's recommendations and administered within 30 min from preparation. None of the subjects included in this study were taking immunosuppressive agents before vaccination. All subjects who participated in this retrospective observational study provided two separate written informed consents for being vaccinated and for inclusion in the post-vaccination anti-SARS-CoV-2 survey. This retrospective observational study was conducted in accordance with the Declaration of Helsinki and its protocol was cleared by the Ethics Committee of the Provinces of Verona and Rovigo (3246CESC).

### Blood sampling and laboratory testing

Venous blood was collected by venipuncture within evacuated blood tubes with clot activator and gel (Greiner Bio-One, Kremsmünster, Austria) at three different time points, i.e., just before the administration of the first vaccine dose, at 21 days (just before the administration of the second vaccine dose) and, finally, 50 days after the first vaccine dose (i.e., 1 month after the second vaccine dose). Serum was separated from blood cells by centrifugation at 1500×g for 15 min at room temperature and separated in two identical aliquots of approximately 1.5 mL, which were keep stored at -70°C until all measurements were performed. The three paired aliquots collected at the three different time points from each participant were concomitantly thawed, re-centrifuged and tested with three different anti-SARS-CoV-2 immunoassays, as summarized in Table 1. These included the assessment of anti-spike trimeric IgG, anti-spike RBD IgG and anti-spike S1 subunit IgA. All three assays were performed in accordance with manufacturer's recommendations.

**Table 1:** Technical characteristics of the anti-SARS-CoV-2 antibodies immunoassays used in this study AU, arbitrary units; BAU, binding antibody units; CLIA, ChemiLuminescent ImmunoAssay; Ig, ELISA; enzyme linked immunoassay; Ig, Immunoglobulin; RBD, Receptor Binding Domain; S1, Spike protein S1 subunit

Immunoassay	Company	Analyzer	Principle	Antibodies	Target	Cut-off
LIAISON SARS-CoV-2 TrimericS IgG	DiaSorin	LIAISON XL	CLIA	IgG	Spike protein trimer	<33.8 BAU/mL
ACCESS SARS-CoV-2 IgG II	Beckman Coulter	Access 2	CLIA	IgG	RBD	<10 AU/mL
Anti-SARS-CoV-2 ELISA IgA	Euroimmun	-	Manual ELISA	IgA	S1	<1.1 ratio

### Statistical analysis

The results of the three anti-SARS-CoV-2 immunoassays are presented as median and interquartile range (IQR), or as ratio with baseline anti-SARS-CoV-2 antibodies value (i.e., (21 or 50 days time point value)/(baseline value and/or limit of detection)). Spearman's test was used to assess correlations between the results of anti-SARS-CoV-2 immunoassays or patient characteristics. The statistical analysis was performed using Analyse-it (Analyse-it Software Ltd, Leeds, UK).

## Results

**Figure 1:**
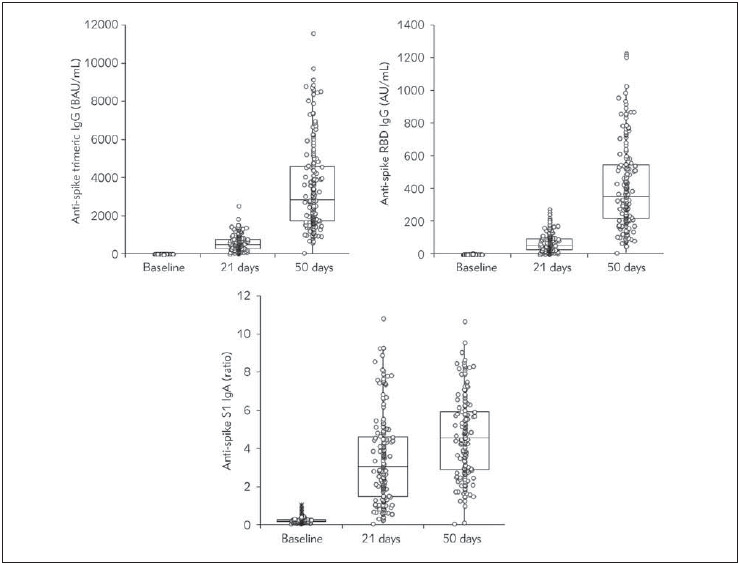
Kinetics of anti-SARS-CoV-2 antibodies development after Pfizer Comirnaty mRNA vaccination Ig, Immunoglobulin; RBD, Receptor Binding Domain; S1, Spike protein S1 subunit

The final study population consisted of 181 SARS-CoV-2 seronegative healthcare workers (median age 42 years, IQR 31-52 years; 108 women, 59.7%; 7 subjects >65 years, 3.9%). The kinetics of anti-SARS-CoV-2 antibodies development after Pfizer Comirnaty vaccination is summarized in Table 2 and Figure 1. Vaccine administration was effective to elicit sustained production of all anti-SARS-CoV-2 antibodies classes. Before administration of the second vaccine dose, positivization occurred for 98.3%, 92.8% and 81.8% cases for anti-spike trimeric IgG, anti-spike RBD IgG and anti-spike S1 IgA, respectively. Such percentages increased to 100% for both anti-spike trimeric IgG and anti-spike RBD IgG 30 days after the second vaccine dose, whilst 1.7% of subjects remained anti-spike S1 IgA negative.

**Table 2:** Kinetics of anti-SARS-CoV-2 antibodies development after Pfizer Comirnaty vaccination AU, arbitrary units; BAU, binding antibody units; Ig, Immunoglobulin; RBD, Receptor Binding Domain; S1, Spike protein S1 subunit

Antibodies	Baseline	21 days	50 days
Anti–spike trimeric IgG			
– Serum values (BAU/mL)	<4.8	504.4 (280.8–770.5)	2834 (1740.3– 4576.0)
– < cut-off (n; %)	181/181 (100%)	178/181 (1.7%)	0/181 (0%)
Anti-spike RBD IgG			
– Serum values (AU/mL)	0.21 (0.12–0.39)	52.3 (26.0–92.7)	350.6 (218.3–543.2)
– < cut-off (n; %)	181/181 (100%)	168/181 (7.2%)	0/181 (0%)
Anti-spike S1 IgA			
– Serum values (ratio)	0.19 (0.15–0.27)	3.06 (1.49–4.62)	4.55 (2.89–5.94)
– < cut-off (n; %)	181/181 (100%)	148/181 (18.2%)	178/181 (1.7%)

**Figure 2:**
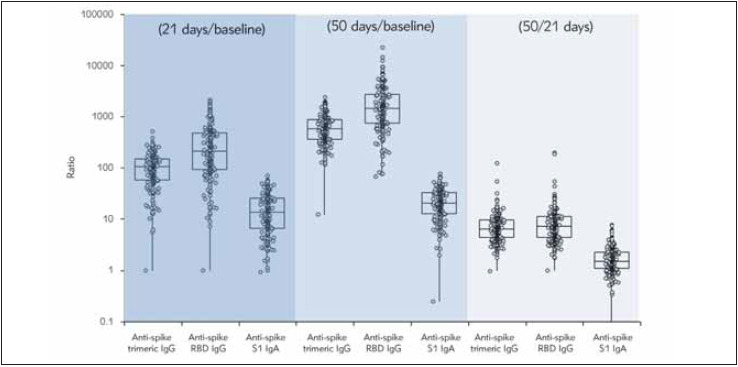
Median increase (ratio) of the different classes of anti-SARS-CoV-2 antibodies elicited by the two Pfizer Comirnaty mRNA vaccine doses Ig, Immunoglobulin; RBD, Receptor Binding Domain; S1, Spike protein S1 subunit

The median increase (ratio) of the different classes of anti-SARS-CoV-2 antibodies elicited by the two Pfizer Comirnaty vaccine doses is shown in Figure 2. In summary, the first vaccine dose triggered a median increase from baseline of 103.3(IQR, 58.2–147.3) folds for anti-spike trimeric IgG, 210.9 (IQR, 90.4–469.5) folds for anti-spike RBD IgG, and 13.3 (IQR, 6.5–25.0) folds for anti-spike S1 IgA, whilst the second vaccine dose elicited a median increase from baseline of 576.1 (IQR, 360.7–867.8) folds for anti-spike trimeric IgG, 1426.0 (IQR, 742.0–2698.6) folds for anti-spike RBD IgG, and 20.2 (IQR, 12.5–32.1) folds for anti-spike S1 IgA. Therefore, compared to the anti-SARS-CoV-2 values attained with the first vaccine dose, the second boost triggered a further modest median increase of these antibodies, being 6.3 (IQR, 4.4–9.5) folds for anti-spike trimeric IgG, 7.2 (IQR, 4.4–11.2) folds for anti-spike RBD IgG, and 1.5 (IQR, 1.1–2.3) folds for anti-spike S1 IgA, respectively. The inter-individual variation (expressed as coefficient of variation; CV%) of anti-SARS-CoV-2 antibodies levels achieved after the first and second vaccine dose was considerably broad, being 70.6% and 65.2% for anti-spike trimeric IgG, 81.2% and 62.0% for anti-spike RBD IgG, and 71.6% and 66.9% for anti-spike S1 IgA, respectively.

**Figure 3:**
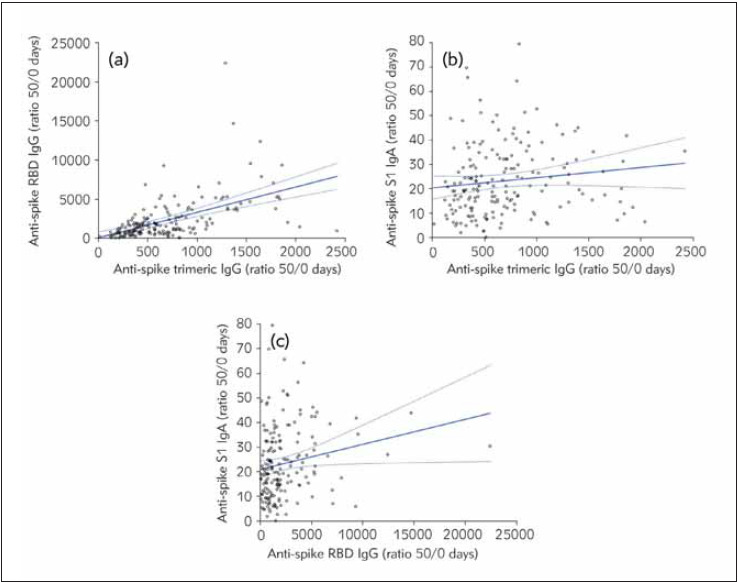
Spearman’s correlation between the overall serum increase of anti-spike trimeric IgG, anti-spike RBD IgG and anti-spike S1 IgA elicited after administration of two Pfizer Comirnaty mRNA vaccine doses. The Spearman’s correlation coefficients were as follows: anti-spike trimeric IgG vs. anti-spike RBD IgG: r=0.60 (95% CI, 0.50–0.68; p<0.001) (Figure 3a); anti-spike trimeric IgG vs. anti-spike S1 IgA: r=0.22 (95% CI, 0.08–0.36; p=0.003) (Figure 3b), and anti-spike RBD IgG vs. anti-spike S1 IgA: r=0.25 (95% CI, 0.11–0.38; p<0.001) (Figure 3c) Ig, Immunoglobulin; RBD, Receptor Binding Domain; S1, Spike protein S1 subunit

Significant Spearman’s correlations were found between the ratios of antibodies levels from baseline up to end of the second cycle of vaccination (i.e., 50 days after the first dose), as follows: anti-spike trimeric IgG vs. anti-spike RBD IgG: r=0.60 (95% CI, 0.50–0.68; p<0.001); anti-spike trimeric IgG vs. anti-spike S1 IgA: r= 0.22 (95% CI, 0.08–0.36; p=0.003), and anti-spike RBD IgG vs. anti-spike S1 IgA: r=0.25 (95% CI, 0.11–0.38; p<0.001) (Figure 3). Notably, a significant inverse association was also found between age and cumulative increase (50 days/baseline ratio) of anti-spike trimeric IgG (r=-0.24; 95% CI, -0.37 to -0.10; p=0.001) and anti-spike S1 IgA (r=-0.16; 95% CI, -0.30 to -0.02; p=0.028), but not with anti-spike RBD IgG (r= -0.05; 95% CI, -0.20 to 0.10; p=0.497), whilst no significant correlation was found between sex and test results any of the three immunoassays (all p>0.05).

## Discussion

With COVID-19 still causing several thousands of deaths every day around the globe, universal equitable access to COVID-19 vaccination seems the key for attenuating the dramatic health, social and economic impact of this ongoing SARS-CoV-2 pandemic outbreak [15]. Preliminary epidemiologic evidence seemingly attests that there is a straightforward association between vaccine distribution and reduction of SARS-CoV-2 positive cases, COVID-19 hospitalizations and deaths [16]. Although it is virtually undeniable that cellular immunity and circulating anti-SARS-CoV-2 IgG antibodies would act in synergy to neutralize the virus either within or outside the host cells [9], mucosal immune response is expected to play a major role in preventing or limiting viral infection, as SARS-CoV-2 initially penetrates the cells of upper and lower respiratory tracts [10]. To this end, the associations between nasal anti-SARS-CoV-2 IgA antibodies responses, virus neutralization at the mucosal surface, and lower risk of developing severe COVID-19 illness provide further support to the clinical significance of assessing and monitoring mucosal immunity in patients with natural SARS-CoV-2 infection and/or in COVID-19 vaccine recipients [17] [18].

Several lines of evidence now attest that the IgA serum levels quite accurately reflect the concentration of these immunoglobulins at the mucosal surface, and especially that mucosal anti-SARS-CoV-2 spike protein IgA levels strongly correlate with virus neutralization potency [19] [20]. As concerns the former aspect, the cumulative seropositivity rate of IgA antibodies targeting the RBD of SARS-CoV-2 is comparable to that of the IgG class, and consistently higher than that of IgM antibodies [21]. Pisanic and colleagues [22] reported that matched serum and saliva anti-SARS-CoV-2 IgA levels measured with many different methods were significantly correlated in patients with COVID-19. A significant correlation between serum and secretory anti-SARS-CoV-2 spike protein IgA (r=0.542; p<0.001) and RBD IgA (r=0.389; p<0.001) has also been reported in COVID-19 patients by Isho and colleagues [23]. These findings would hence convincingly support the hypothesis that serum assessment of anti-SARS-CoV-2 neutralizing antibodies may reliably mirror their secretory counterpart, thus reflecting virus neutralizing potential at the mucosal surface.

With respect to the role played by anti-SARS-CoV-2 IgA antibodies in preventing viral infection, Sterlin et al. [21] showed that the early neutralizing antibody potential seems to be predominated by antibodies of the IgA class, whose appearance is comparable to that of IgG antibodies, but seems significantly earlier than that of antibodies of the IgM class. Importantly, the presence of anti-SARS-CoV-2 RBD IgA in saliva appeared to be strongly correlated with their neutralizing potency. A highly significant correlation between the serum IgA titer and that of anti-SARS-CoV-2 neutralizing antibodies has also been demonstrated by Varnait et al. [24], thus confirming that this class of secretory immunoglobulins may be really effective to neutralize the virus at mucosal surface. Importantly, Quinti et al. [25] recently underlined that special consideration should be given to the fact that the risk of being infected by SARS-CoV-2, developing severe COVID-19 illness or displaying prolonged viral shedding, as well as that of experimenting vaccination failure, may be especially magnified in subjects with insufficient serum and/or secretory anti-SARS-CoV-2 IgA response.

As concerns the specific post-vaccination kinetics of serum neutralizing IgA antibodies compared to that of IgG, our data showed that Pfizer COVID-19 mRNA vaccine administration elicited a very sustained response of anti-spike trimeric IgG (576-fold from baseline) and anti-spike RBD IgG (1426-fold from baseline), whilst the increase of anti-spike S1 IgA was apparently weaker (around 20-fold from baseline), yet detectable and likely of clinical significance. Notably, the first vaccine dose left a considerable number of recipients with negative anti-spike S1 IgA levels (i.e., approximately 18%), whilst the rate of non-responders to the mRNA vaccine decreased to <2% after the second dose (i.e., 3 subjects, two men aged 40 and 58 years, and one women aged 52 years – studies are ongoing to identify the possible reasons). This is congruent with real-life data on the relatively lower efficacy of administering a single mRNA vaccine dose, since vaccine efficiency for preventing SARS-CoV-2 infection was shown to increase from around 46% between 14-20 days after the first vaccine dose, up to 92% more than 7 days after the second vaccine dose [26]. Moreover, vaccine efficacy for preventing COVID-19 symptomatic illness was significantly increased after the second dose (i.e., 57% to 94%), with a similar but more marginal effect on hospitalizations (i.e., 74% to 87%) [26]. Taken together, this data strongly suggests that the lower anti-SARS-CoV-2 neutralizing IgA antibodies response observed in our study after a single mRNA vaccine dose would actually mirror a still insufficient mucosal protection against SARS-CoV-2, thus corroborating current beliefs that the second mRNA vaccine dose is essential for achieving a sufficient level of herd immunity [27], which may hence at least partially develop through an adequate increase of serum and mucosal levels of anti-SARS-CoV-2 IgA neutralizing antibodies. Another important aspect that has emerged from our study, is that the relative increase from baseline of anti-spike S1 IgA levels was inversely correlated with age, as it was the increase of anti-spike trimeric IgG, so that consideration shall be given to more strictly monitoring both antibodies classes in older people and eventually define a personalized vaccination program (i.e., using a third booster).

In conclusion, the results of our study attest that mRNA COVID-19 vaccination is effective to elicit sustained levels of serum anti-spike trimeric IgG and anti-spike RBD IgG, while also modestly but significantly increasing those of serum anti-spike S1 IgA.

*Research funding*: None declared.

*Author contributions*: All authors have accepted responsibility for the entire content of this manuscript and approved its submission.

*Informed consent*: Informed consent was obtained from all individuals included in this study.

*Ethical approval*: The study was cleared by the Ethics Committee of the Provinces of Verona and Rovigo (3246CESC).

## Conflict of interest statement

All the authors declare that they have no conflict of interest in this work.
